# Quantification and Diversity Analyses of Major Glucosinolates in Conserved Chinese Cabbage (*Brassica rapa* L. ssp. *pekinensis*) Germplasms

**DOI:** 10.3390/foods12061243

**Published:** 2023-03-14

**Authors:** Seong-Hoon Kim, Gi-An Lee, Parthiban Subramanian, Bum-Soo Hahn

**Affiliations:** 1 National Agrobiodiversity Center, National Institute of Agricultural Sciences, RDA, Jeonju 5487, Republic of Korea; 2 Department of Physiology, Saveetha Dental College & Hospitals, Saveetha Institute of Medical and Technical Sciences (SIMATS), Saveetha University, Chennai 600 077, Tamil Nadu, India

**Keywords:** Chinese cabbage, glucosinolates, germplasm, diversity

## Abstract

The genebank at the National Agrobiodiversity Center (RDA-Genebank, Jeonju, Republic of Korea), conserves approximately 8000 germplasms of *Brassica* spp., of which Chinese cabbage (*Brassica rapa* L. ssp. *pekinensis*) is one of the major crops actively used as food in Northeast Asia, including Korea, as the main ingredient for kimchi. Glucosinolates are a major class of compounds in Chinese cabbage that are responsible for their distinctive flavor, and RDA-Genebank is constantly building a related database (DB) to select suitable germplasms required by consumers and provide resources for breeding programs. In this study, ten glucosinolates were analyzed in sixty Chinese cabbage germplasms. Six aliphatic glucosinolates were the major components, accounting for 85.00% to 91.98% of total glucosinolates in each germplasm. Among them, gluconapin (333.26 to 23,501.58 μmol∙kg^−1^ DW) was highly represented, followed by glucobrassicanapin (545.60 to 10,344.70 μmol∙kg^−1^ DW) and progoitrin (155.28 to 8536.51 μmol∙kg^−1^ DW). In addition, we selected germplasms with a high content of each studied glucosinolate. To analyze the diversity and distribution of glucosinolates among the studied germplasms, Pearson’s correlation was performed, and the related results were interpreted through their biosynthetic pathways. The k-means clustering indicated four optimal clusters, which were confirmed through principal component analysis. Orthogonal projection to latent structure discriminant analysis (OPLS-DA) was also performed on the status (landrace and cultivar) and origin (Korea, China, Taiwan, and Japan) passport data of the germplasms, followed by the calculation of variable importance in the projection (VIP) values. These results are part of a continuous series of studies to analyze the glucosinolates of Brassica germplasms that are being conserved at RDA-Genebank. We aim to provide related results through a public platform accessible to everyone and thereby improve the distribution of *Brassica* germplasms.

## 1. Introduction

The Brassicaceae family, also known as the Cruciferae, includes 375 genera encompassing 3200 species [1]. Depending on the crop, various parts of the Brassicaceae plants, including the roots, stems, flower stack, leaves, and seeds, have been used as vegetables, condiments, spices, and medicine [2]. The genus *Brassica* is the largest and most important genera of the family, historically providing multitude of applications to mankind. Humans have used *Brassica* as fresh or cooked food, fodder, forage, a source of nutrients, medicinal compounds, and edible oil, as condiments, for illumination, lubricants, and even to improve soil quality [3]. Historically, members of the genus Brassica have been valued and cultivated by the Romans, Greeks, Chinese, and Indians for several centuries and have provided a broad array of useful products to humans despite ascending from a single genus [3,4]. The anthropogenic association of the members of this genus has a long history dating back to the 1700s, and they are expected to have greater potential even in the future [5]. Among Brassica species, Chinese cabbage (*Brassica rapa* L. ssp. *pekinensis*) is used as the main ingredient of fermented food kimchi and remains an integral part of Korean cuisine; it is served with almost every meal in Korea [6]. Brassicaceae plants are also well-known for their naturally pungent components, which give them their characteristic smell and taste. One of the major compounds produced by plants of the Brassicaceae family are glucosinolates (GSLs). Glucosinolates are organosulfur compounds that are secondary metabolites produced by plants from glucose and amino acids. These compounds can serve as sulfur storage molecules, help in plant defense, protect against pests, and provide the characteristic pungent flavor to cruciferous vegetables and their products.

In recent times, bitterness has become an important criterion in animal food preferences [7]. In the case of Chinese cabbage glucosinolates, gluconapin and glucobrassicanapin are known to be directly related to its characteristic bitter taste [8]. In broccoli, the GSL progoitrin is known to be involved in bitterness as well as to affect growth retardation in some animals [9]. For this reason, many studies on glucosinolate hydrolysis products, including isothiocyanates, epithionitriles, and nitrile, have been conducted. Among the hydrolysis products, isothiocyanates have a direct positive effect on human health. Some isothiocyanates activate phase 2 detoxification enzymes, resulting in the inhibition of cancer growth and carcinogens [10]. Additionally, in Chinese cabbage, it is known that glucosinolate degradation products play a role in defense against other insects [11].

**Figure 1:**
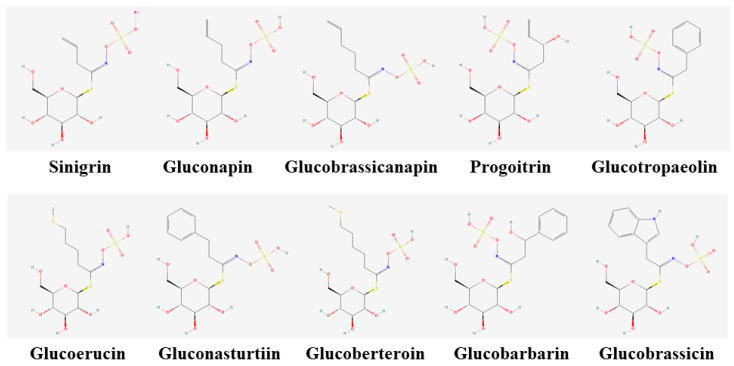
Chemical structures of ten glucosinolates were analyzed in this experiment.

According to their chemical structure, GSLs can be classified into three groups: aliphatic, aromatic, and indolic [12], depending on the amino acid precursors in their side chains: methionine, tryptophan, and aromatic amino acids (tyrosine or phenylalanine), respectively (Figure 1) [13]. Among the most commonly found glucosinolate compounds, aliphatic glucosinolates, glucoiberverin, glucoiberin, and sinigrin contain three carbons in their chain; glucoerucin, dehydroerucin, glucoraphanin, glucoraphenin, gluconapin, progoitrin contain four carbons; and glucoberteroin, glucoalyssin, glucobrassicanapin, gluconapoleiferin are made of five carbons in their chain. Gluconasturtiin is an aromatic glucosinolate, followed by glucobrassicin, 4-hydroxyglucobrassicin, 4-methoxyglucobrassicin, and neoglucobrassicin, which are indolic glucosinolates. In the past, aliphatic glucosinolates derived from methionine were considered important [14], but now, as research continues, other important glucosinolates are being discovered. Major international plant gene banks such as ARS in the United States, IPK in Germany, VIR in Russia, NARO in Japan, and RDA-Genebank in Korea recognize the importance of plant genetic resources and are making every effort to collect, conserve, and evaluate plants. With such vast upcoming therapeutic uses for glucosinolates, it is essential to characterize the available bioresources conserved in plant gene banks for suitable applications in the future. In the current study, sixty Chinese cabbage (*Brassica rapa* L. ssp. *pekinensis*) germplasms conserved at the National Agrobiodiversity Center (RDA-Genebank), South Korea, were studied for their glucosinolate profiles. Improving the passport data of such accessions conserved at plant gene banks will be potentially helpful to researchers and breeders in the selection of appropriate germplasms according to the targets of research/breeding. 

## 2. Materials and Methods

### 2.1. Glucosinolate Standards 

All the chemicals used for extraction as well as analyses in this experiment were analytical-grade products from Fisher Scientific Korea Ltd. (Seoul, Republic of Korea) and Sigma-Aldrich (St. Louis, MO, USA). In addition, ten commercially available standard glucosinolates (sinigrin, progoitrin, gluconasturtiin, glucobarbarin, glucobrassicin, glucobrassicanapin, glucoberteroin, glucoerucin, gluconapin, and glucotro-paeolin) were obtained from Phytoplan Diehm & Neuberger GmbH (Heidelberg, Germany) and have a purity of ≥97%. 

### 2.2. Brassica Germplasms and Growth Condition

In this study, sixty Chinese cabbage germplasms were randomly selected among 2800 conserved at the National Agrobiodiversity Center (RDA-Genebank) of the Rural Development Administration (RDA), Jeonju, Republic of Korea [15]. The collected germplasms were from five countries, namely (in order of number): Korea (32), Taiwan (11), China (10), Japan (6), and Thailand (1). With regards to their status, they were spread as cultivars (32) and landraces (25), and germplasms without information were classified as unknowns (3). In order to maintain purity, the *Brassica* germplasms were harvested in the greenhouse from January to July 2020, removing heterogeneous germplasms within the accessions to obtain the same phenotype. The collected seeds were cultivated in the open field again in the fall of 2020, and the germplasms confirmed to have the same phenotype were selected and used as materials for this experiment.

### 2.3. Sample Pretreatment and Extraction

Leaves from random plants in each accession were harvested, collected in vinyl bags, and then temporarily stored at −80 °C. Next, the leaf samples were lyophilized using a LP500 vacuum freeze-drier (Ilshinbiobase Co., Dongducheon, Republic of Korea) for 48 h, following which they were ground into a fine powder. Samples were moved again to −80 °C until analysis. The extraction of GSLs from samples was carried out following the methods established earlier [16]. Briefly, to 0.1 g of sample, 5 mL of 80% methanol was added, and the mixture was kept at 25 °C for 30 min, following which it was shaken at 120 rpm for 30 min at room temperature. The mixture was centrifuged (14,000 rpm at 4 °C for 10 min) and the supernatants were transferred to clean vials for further analysis.

### 2.4. Identification and Quantification of GSLs Using UPLC-MS/MS

The Acquity UPLC System (Waters, Milford, CT, USA) connected to the Xevo™ TQ-S system (Waters, MS Technologies, UK) was used to analyze GSLs according to Rhee et al. 2020 protocols. The separation of 5 µL samples was performed on an Acquity UPLC BEH C18 (1.7 µm, 2.1 × 100 mm) column (Waters Corp., UK) with 0.1% tri-fluoroacetic acid in water as eluent A and 0.1% trifluoroacetic acid in methanol as eluent B mobile phase at a flow rate of 0.5 mL/min and 35 °C. The elution conditions were set at 100% of A from 0.0 to 1.0 min, 100% of A from 1.0 to 7.0 min, 100 to 80% of A from 7.0 to 10 min, 80 to 0% of A from 10 to 11 min, 0 to 100% of A from 11 to 15 min, and 100% of A thereafter. The MS/MS in negative ion electrospray ionization (ESI) and multiple reaction monitoring (MRM) modes were used for detecting GSLs, and capillary and con voltages of 3 kV and 54 V, respectively, were set for ionization. The detected GSLs were identified by comparing their retention times and MS and MS/MS fragmentation spectra with those of commercially procured standards. 

The method’s precision and accuracy were validated by measuring the linear, intraday, and interday precision. For the preparation of standards, 10 mg of individual glucosinolates in methanol were dissolved to obtain stock solutions (1 mg mL^−1^). Calibration curves plotted using the corresponding standards were used for the calculation of GSL concentrations, and the results were represented as µmol GSLs kg^−1^ sample dry weight (DW). The limit of detection (LOD) as well as the limit of quantification (LOQ) values were determined, respectively, as three and ten times the standard error of the intercept of the regression equation of the linear calibration curve divided by the slope. Fresh test solutions were always prepared before sample analysis [17] (Table 1). 

### 2.5. Statistical Analysis

To prove that each variable is independent of the others, the Bartlett sphericity test was applied [18]. The rationality of the data structure of the results of this experiment was confirmed through the Kaiser–Meyer–Olkin (KMO) test [19]. 

To analyze the diversity of the GSLs between germplasms, the methods of Kim et al., (2022) were applied [20,21]. XLSTAT software version 2019 (Addinsoft, Paris, France) was used to select three principal components with an eigenvalue of one or more and was also used in the Pearson’s correlation. Principal component analysis (PCA), the cluster dendrogram, and orthogonal partial least squares discriminant analysis (OPLS-DA) results were carried out using SIMCA (ver.13.3; Umetrics, Sweden).

## 3. Results and Discussion

### 3.1. Variation of GSL Contents and Selection of Candidate Germplasms for the Breeding Program

In this study, Chinese cabbage germplasms conserved at the National Agrobiodiversity Center (RDA-Genebank) were studied using UPLC-MS/MS for their glucosinolate distribution and contents (GSLs). In the sixty studied germplasms, all belonging to *Brassica rapa* L. ssp. *pekinensis,* ten glucosinolate compounds were identified, of which six were aliphatic (sinigrin, gluconapine-GNA, glucobrassicanapin-GBN, progoitrin-PRO, glucoerucin, and glucoberteroin), three aromatic (glucotropaeolin, gluconasturtiin-GNS, and glucobarbarin), and one indolic (glucobrassicin- GBS). 

The contents of glucosinolates in the sixty Chinese cabbage germplasms are given in Table 2. Aliphatic GSLs were the predominant GSL group observed in Chinese cabbage, ranging from 85.00 to 91.98% of the total glucosinolate content (not shown). Among aliphatic GSLs, the GNA (333.26 to 23,501.58 μmol∙kg^−1^ DW) was highly represented, followed by the GBN (545.60 to 10,344.70 μmol∙kg^−1^ DW) and the PRO (155.28 to 8536.51 μmol∙kg^−1^ DW). Among the aromatic GSLs, Gluconasturtiin (GNS) was the most predominant, ranging from 109.48 to 1494.47 μmol∙kg. In a recent study, Gluconasturtiin-Isothiocyanate (ITC), a hydrolysis product by myrosinase, induces apoptosis in human hepatocarcinoma HepG2 cells and human breast adenocarcinoma MCF-7 cells and is recognized for its potential in cancer treatment [22]. The content of GBS, the only indolic GSL detected in this study, ranged from 72.89 to 2213.95 μmol∙kg^−1^ DW. The decomposition byproducts of indole glucosinolates, such as GBS, have been recognized as phytoalexins in several species of *Brassica*, and they are acknowledged to have a significant role in plants defense metabolism [23]. In addition, sulforaphane, indole-3-carbinol, and glucobrassin (GBS), produced by myrosinase, are known to have anticancer functions [10].. They inhibit phase 1 detoxification enzyme activity and induce the activity of phase 2 detoxification enzymes to suppress cancer development [24,25].

GSLs are secondary metabolites in *Brassica* species, and the major GSLs vastly vary across different crops. For example, in the case of broccoli (*B. oleracea* var. *italica*), glucoraphanin remains the major GSL, and in leaf mustard (*B. juncea*), it is sinigrin. Whereas, in the current study, the major GSLs in Chinese cabbage (*B. rapa* ssp. *pekinesis*) have been found to be gluconapine (GNA) and glucobrassicanapine (GBN). In a previous study on Korean cabbage leaves, the estimated GNA and GBN were in a range of 400 to 8990 μmol∙kg^−1^ DW and 490 to 8080 μmol∙kg^−1^ DW, respectively [9], and the analysis results of Chinese cabbage leaves were from 21 to 13,634 μmol∙kg^−1^ DW and 1 to 19,700 μmol∙kg^−1^ DW, respectively [17]. In particular, Park et al. (2019) [26] reported gluconapine (GNA) in a commercial Chinese cabbage cultivar (Miss jin), which was 20,456 μmol∙kg^−1^ DW. Commercial cultivars have high GNA and GBN, but they also have a high PRO of 1770~6070 μmol∙kg^−1^ DW, which causes bitterness and reduces appetite [7]. Leaves or parts of plants that contain PROs in a range of 2300 to 4650 μmol∙kg^−1^ DW are not preferred by animals when used as feed [27]. 

In the current study, we selected germplasms that contained high GSL content, with a range of over 10,000 μmol∙kg^−1^ DW GNA. The germplasm IT100355 (AVRDC-KJH-1985-100355) produced the highest amounts of GNA with 23,501 μmol∙kg^−1^ DW, while IT100353 (AVRDC-KJH-1985-100353) and IT100354 (AVRDC-KJH-1985-100354) were at 14,332 μmol∙kg^−1^ DW and 12,686 μmol∙kg^−1^ DW, respectively. Of these, IT105353 had the third highest GBN content at 9803 μmol∙kg^−1^ DW. However, due to the bitter taste of PRO 3446 μmol∙kg^−1^ DW, its use could be limited to breeding programs. In this experiment, we recommend breeding three germplasms that are characterized by high GNA and GBN tendencies while producing low PRO content and, thereby, less bitter. The first recommended material was IT32753 (wonsi-1984), with GNA and GBN content at 9797 μmol∙kg^−1^ DW and 10,344 μmol∙kg^−1^ DW, respectively, while PRO was relatively low at 2667 μmol∙kg^−1^ DW. Next, IT100355 (AVRDC-KJH-1985-100355) had a GNA and GBN of 23,501 μmol∙kg^−1^ DW and 6743 μmol∙kg^−1^ DW, respectively, while PRO content was estimated to be 2388 μmol∙kg^−1^ DW. The last germplasm was IT32755; GNA and GBN were 8142 μmol∙kg^−1^ DW and 8779 μmol∙kg^−1^ DW, respectively, while PRO was found to be at 1870 μmol∙kg^−1^ DW.

### 3.2. Correlation Analysis among the GSLs

**Figure 2:**
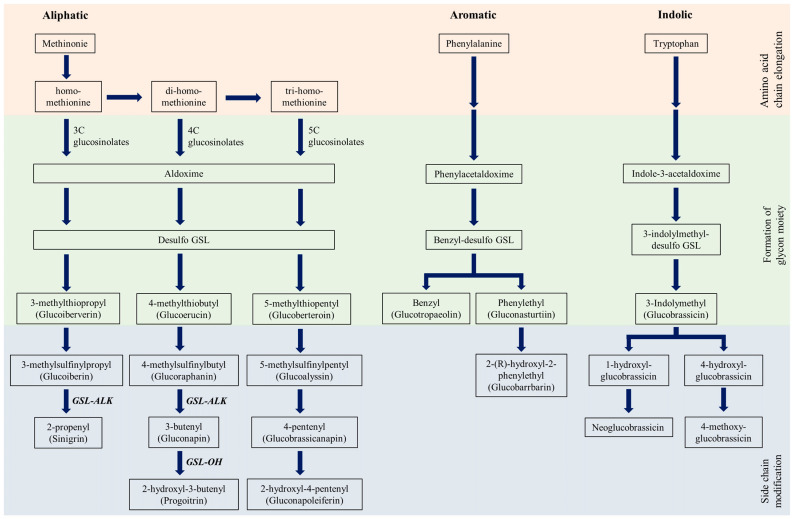
Three major biosynthesis pathways of glucosinolate in Brassicaceae. Flow chart, which can be divided into three pathways: amino acid chain elongation, formation of the glycon moiety, and side chain modification. Texts in bold italics indicate the biosynthetic genes involved in each step.

To investigate the relationship between glucosinolate compounds, we performed Pearson’s correlation analysis (Table 3). A significant correlation between gluconapin (GNA) and glucobrassicanapin (GBN), known as major glucosinolates in Chinese cabbage, was positively confirmed (*r* = 0.652, *p* < 0.00.1). In the aliphatic GSL synthesis pathway, progoitrin (PRO) and gluconapin (GNA), which are precursors of progoitrin (PRO), were positively correlated (*r* = 0.531, *p* < 0.001). The varied biosynthetic pathways for different glycosinolates corroborate with these results, and commonly aliphatic glucosinolates were found to be positively correlated (Figure 2). In the aromatic GSL synthesis pathway, a positive correlation was confirmed between gluconasturtiin (GNS), the precursor of glucobarbarin (GBB), and glucobarbarin (GBB) (*r* = 0.430, *p* < 0.01). Furthermore, as expected from the aromatic GSL synthesis pathway, gluconasturtiin (GNS) and glucotropaeolin (GTL), which diverged from the synthesis pathway, were found to have negative correlations with each other (*r* = −0.238, *p* < 0.05). Glucoraphanin, a major GSL in broccoli, is decomposed by myrosinase and converted to sulforaphane, which is widely known to suppress the occurrence of prostate [28] and lung cancer [29]. Lee et al. (2012) [30] suggested that the correlation between glucoraphanin and progoitrin (PRO) was negative. Although glucoraphanin was not analyzed in this experiment, glucoraphanin (GBN) and progoitrin (PRO), which are involved in the chain elongation process in the aliphatic GSL synthesis pathway, have differences in the enzyme activity of GLS-ALK or GSL-OH due to genetic differences among germplasms [31]. In order to select high-content glucoraphanin germplasms, a molecular breeding strategy that suppresses conversion to progoitrin (PRO) or gluconapin (GNA), which are lower-level substances, is required (Figure 2). 

### 3.3. Clustering and Diversity Analysis

**Figure 3:**
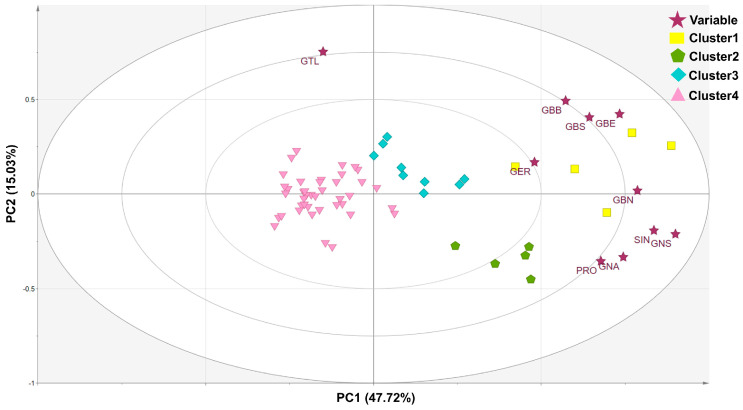
Results of principal components 1 (47.72%) and 2 (15.03%) with 60 germplasms based on ten glucosinolates. The score plots were represented by yellow, green, blue, and pink colors. The star shapes describe loading plots.

The KMO test coefficient for sample adequacy and Bartlett’s test of sphericity were observed to be greater than 0.6, which indicated that the sample met the requirements of a reasonable data structure. Principal component analysis (PCA) is one of the most popular clustering methods and is widely used to show the largest variance within a population and to determine the most relevant components [32]. In our experiments, the PCA was performed to determine the differences among 60 germplasms in ten GSL content profiles. Each data point on the score plot (Figure 3) shows individual germplasm, and each point on the loading plot shows the contribution of an individual GSL to the score plot [33].

The three principal components were selected with eigenvalue ≥1, explaining 73.90% of the total germplasms (Table 4). In the principal component (PC), it was shown that the first, second, and third PC explained 46.72, 15.03, and 12.15% of the total germplasm, respectively. The PC1 was influenced by GNS, SIN, GBN, GNA, and GBE. The glucosinolates GTL and GBB were identified as major variations determined by PC2. Whereas, the PC3 had high positive values for the GER and negative values for the GBN (Table 4). 

**Figure 4:**
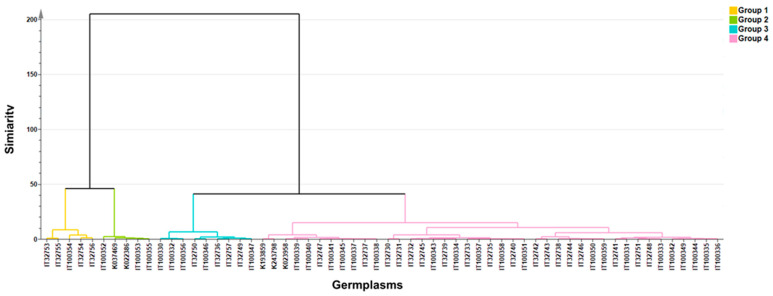
Dendrogram from results of the k-means clustering. The four cluster groups are indicated by yellow, green, blue, and pink colors.

As a result of hierarchical cluster analysis, the sixty Chinese cabbages were classified into four groups based on the contents of GSLs (Figure 4). The GTL, which was positively loaded to PC2, was negatively loaded to PC1. Among GSLs positively loaded to PC1, the GBN, GER, GBE, GBB, and GBS were positively loaded to PC2, on the other hand, the SIN, GNA, PRO, and GNS were negatively loaded to PC2. PCA plots were categorized into four groups based on hierarchical clusters. Cluster 4 is located in the middle-left quadrant, with negative PC1 values. Cluster 1 and cluster 3 were distributed in the upper-right quadrant, representing positive values for PC1 and PC2. Cluster 2 was clustered in the lower-right quadrant, with a positive PC1 value and a negative PC2 value. 

Table 5 shows the status and origin information of germplasms by cluster. Many studies have reported on the glucosinolate content of plants from the genus Brassica, including Chinese cabbage. However, international gene banks can provide a broad picture of information on plants, which may include bioactive components, origin and other passport information, and even phenomic data, often at a very large scale. 

**Figure 5:**
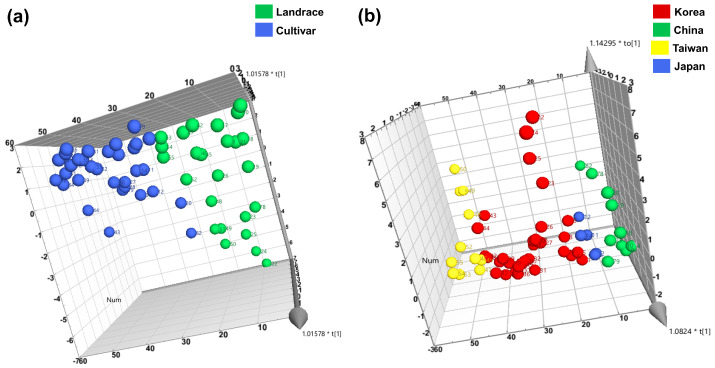
3D Orthogonal projections to latent structure discriminant analysis (OPLS-DA) score plots of Chinese cabbage germplasms. The score plot was clustered based on status (cultivar or landrace) and origin (Korea, China, Taiwan, and Japan). Clustered into two statuses (**a**); and four origins (**b**); respectively.

**Figure 6:**
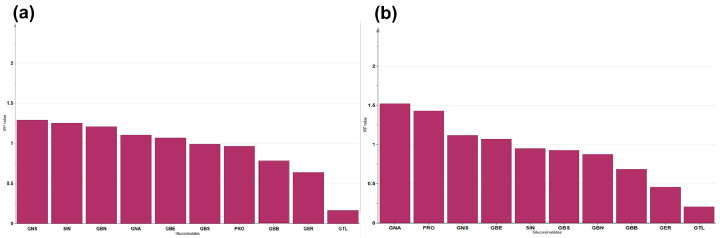
Variable importance in the projection (VIP) values linked with OPLS-DA clustered based on ten glucosinolates. Variable shown in order of major contribution to two statuses (**a**); and four origins (**b**).

Orthogonal partial least squares discriminant analysis (OPLS-DA) clustered 10 GSLs into origin and status (cultivar and landrace), respectively (Figure 5). In addition, the major GSLs contributing to the distinction between clusterings were identified by applying the variable influence on the projection (VIP) value (Figure 6). As shown in Figure 5, the germplasms were classified into two groups: landraces and cultivars (Figure 5a). Based on VIP value one or higher, the five GSLs (in order of importance: GNS, SIN, GBN, GNA, and GBE) were the variables contributing to the separation between clusters, and in particular, GNS, SIN, and GBN were major variables (Figure 6a). Figure 5b shows that the results of classification by origin are also clearly divided into four clusters. GBB, GER, and GTL contributed little to the clustering of status and origin, and GTL in particular had little effect on the clustering results (Figure 6b). We plan to continuously analyze individual glucosinolates for Brassica subspecies, which are conserved in RDA-Genebank, and determine whether they can be classified based on passport data parameters, including status and origin. 

## 4. Conclusions

In this study, sixty Chinese cabbage (*Brassica rapa* L. ssp. *pekinensis*) germplasms conserved in RDA-Genebank were studied for their GSL content in leaves using UPLC-MS/MS. For Chinese cabbage, gluconapin (GNA) and glucobrassicanapin (GBN) are known as major glucosinolates, and the results of this study were also consistent with the results of the previous study. Pearson’s correlation of the ranges of different GSLs detected in this study was consistent with the GSL biosynthetic pathways in plants. We also grouped the sixty germplasms into four clusters using k-means clustering, which was later confirmed through PCA. 

In particular, by applying OPLS-DA to germplasm status and origin, the main contributing glucosinolates were identified as VIP values. 

A germplasm with high GNA and GBN but low PRO was selected and recommended here as a material for a breeding program. We plan to continuously analyze GSLs for Brassica species being conserved at RDA-Genebank and share related information so that all consumers can utilize it.

## Figures and Tables

**Table 1:** UPLC spectroscopy information on ten glucosinolates studied in sixty Chinese cabbages.

Name	Abbreviation	Group	RT (min)	MRM Transition	CID (ev)	Dwell Time (sec)	Calibration Curve Parameters
**Progoitrin**	PRO	Aliphatic	5.96	387.77 > 194.85	25	0.029	Y = 8.2526X + 28.1501 (r^2^ = 0.962)
**Sinigrin**	SIN	Aliphatic	6.55	357.75 > 161.84	25	0.029	Y = 12.7878X − 11.1181 (r^2^ = 0.999)
**Gluconapin**	GNA	Aliphatic	7.77	371.74 > 258.74	25	0.029	Y = 8.36216X + 29.5397 (r^2^ = 0.994)
**Glucobrassicanapin**	GBN	Aliphatic	8.6	385.71 > 258.87	25	0.029	Y = 7.2514 X + 47.2841 (r^2^ = 0.992)
**Glucobarbarin**	GBB	Aromatic	8.65	437.71 > 274.75	25	0.029	Y = 9.29915 X − 0.454779 (r^2^ = 0.999)
**Glucoerucin**	GER	Aliphatic	8.76	419.69 > 258.74	25	0.029	Y = 6.77393 X + 73.6679 (r^2^ = 0.985)
**Glucotropaeolin**	GTL	Aromatic	8.88	407.72 > 258.87	25	0.029	Y = 18.2122X − 3.93949 (r^2^ = 0.999)
**Glucoberteroin**	GBE	Aliphatic	9.18	433.72 > 275.06	25	0.029	Y = 6.09397 X + 63.1212 (r^2^ = 0.971)
**Glucobrassicin**	GBC	Indolyl	9.32	446.69 > 204.94	25	0.029	Y = 6.39827 X + 2.6232 (r^2^ = 0.998)
**Gluconasturtiin**	GNS	Aromatic	9.36	421.69 > 274.87	25	0.029	Y = 4.36109 X + 90.233 (r^2^ = 0.961)

**Table 2:** Profile of individual glucosinolates in 60 kimchi cabbage germplasm samples (μmol∙kg^−1^ DW).

Variable	SIN	GNA	GBN	PRO	GTL	GER	GNS	GBE	GBB	GBS
Minimum	0.30	333.26	545.60	155.28	0.55	0.26	109.48	2.15	ND	72.89
Maximum	20.00	23,501.58	10,344.70	8536.51	40.77	207.29	1494.47	2109.97	150.69	2213.95
Mean	3.42	3432.88	4065.17	1740.44	12.49	28.18	514.58	248.18	49.82	633.95
Std. deviation	4.36	4088.64	2616.67	1499.76	7.45	39.00	336.82	387.90	33.79	490.51

ND—not detected. Glucobarbarin (GBB), glucoberteroin (GBE), glucobrassicanapin (GBN), glucobrassicin (GBS), glucoerucin (GER), gluconapin (GNA), gluconasturtiin (GNS), glucotropaeolin (GTL), progoitrin (PRO), and sinigrin (SIN).

**Table 3:** Pearson’s correlation analysis between glucosinolate compounds.

	SIN	GNA	GBN	PRO	GTL	GER	GNS	GBE	GBB
GNA	0.625 ***								
GBN	0.759 ***	0.652 ***							
PRO	0.502 ***	0.531 ***	0.358 ***						
GTL	−0.217	−0.227	−0.010	−0.203					
GER	0.210	0.183	0.038	0.382 **	−0.122				
GNS	0.734 ***	0.683 ***	0.627 ***	0.756 ***	−0.238 *	0.424 **			
GBE	0.544 ***	0.312 *	0.481	0.272 *	0.015	0.573 ***	0.529 ***		
GBB	0.224	0.345 *	0.443	0.247	0.195	0.343	0.430 *	0.540 ***	
GBS	0.531 ***	0.274 *	0.516 ***	0.243	0.144	0.224	0.466 *	0.601 ***	0.347 **

*, **, *** Correlationship is significant at *p* ≤ 0.05, 0.01, and 0.001, respectively. Glucobarbarin (GBB), glucoberteroin (GBE), glucobrassicanapin (GBN), glucobrassicin (GBS), glucoerucin (GER), gluconapin (GNA), gluconasturtiin (GNS), glucotropaeolin (GTL), progoitrin (PRO), and Sinigrin (SIN).

**Table 4:** Three principal components among ten glucosinolates of 60 germplasms.

Glucosinolates	Eigenvector of Principal Component		
	1	2	3
Sinigrin	0.386	−0.157	−0.230
Gluconapin	0.344	−0.271	−0.184
Glucobrassicanapin	0.364	0.015	−0.447
Progoitrin	0.314	−0.289	0.205
Glucotropaeolin	−0.070	0.615	−0.257
Glucoerucin	0.222	0.139	0.718
Gluconasturtiin	0.416	−0.172	0.071
Glucoberteroin	0.340	0.345	0.214
Glucobarbarin	0.265	0.403	0.088
Glucobrassicin	0.298	0.332	−0.176
Eigenvalue	4.67	1.50	1.22
Variability (%)	46.72	15.03	12.15
Cumulative (%)	46.72	61.74	73.90

**Table 5:** Information status and origin in germplasms divided by four clusters.

Clustering	Status	Origin
Cluster 1 (*n* = 5)	Landrace 5	KOR 4, TWN 1
Cluster 2 (*n* = 5)	Landrace 4, Cultivar1	TWN 3, CHN 2
Cluster 3 (*n* = 9)	Cultivar 6, Landrace 3	KOR 6, CHN 2, TWN 1
Cluster 4 (*n* = 41)	Cultivar 25, Landrace 13, Unknown 3	KOR 20, CHN 6, TWN 6, JPN 5, THA 1

KOR—South Korea; TWN—Taiwan; CHN—China; JPN—Japan; THA—Thailand.

## Data Availability

The data within the communication are available.
